# Prevention and Treatment of Experimental Estrogen Receptor – Negative Mammary Carcinogenesis by the Synthetic Triterpenoid CDDO-Methyl Ester and the Rexinoid LG100268

**DOI:** 10.1158/1078-0432.CCR-08-0040

**Published:** 2008-07-15

**Authors:** Karen Liby, Renee Risingsong, Darlene B. Royce, Charlotte R. Williams, Mark M. Yore, Tadashi Honda, Gordon W. Gribble, William W. Lamph, Nicola Vannini, Ilaria Sogno, Adriana Albini, Michael B. Sporn

**Affiliations:** 1 Department of Pharmacology, Dartmouth Medical School; 2 Department of Chemistry, Dartmouth College, Hanover, New Hamsphire; 3 Ligand Pharmaceuticals, Inc., San Diego, California; 4 Multimedica IRCCS, Milan, Italy

## Abstract

**Purpose:**

To test whether the triterpenoid 2-cyano-3,12-dioxooleana-1,9(11)-dien-28-oic acid methyl ester (CDDO-Me) and the rexinoid LG100268 (268) prevent the formation of estrogen receptor (ER) – negative mammary tumors or either arrest the growth or cause regression of established tumors in MMTV-neu mice.

**Experimental Design:**

For prevention, mice were fed control diet, CDDO-Me (60 mg/kg diet), 268 (20 mg/kg diet), or the combination for 45 weeks. For treatment, mice with established tumors at least 4 mm in diameter were fed control diet, CDDO-Me (100 mg/kg diet), 268 (60 mg/kg diet), or the combination for 4 weeks.

**Results:**

CDDO-Me and 268 significantly delayed the development of ER-negative tumors, with a 14- and 24-week delay, respectively, compared with the control group for the time required to reach 50% tumor incidence. The combination of CDDO-Me and 268 was significantly more potent than the individual drugs, as only one tumor was found in the combination group, after 45 weeks on diet, at which time all control animals had tumors. Treating established tumors with CDDO-Me arrested the growth of 86% of the tumors, and 268 induced tumor regression in 85% of tumors. CDDO-Me and 268 target different signaling pathways and cell types. CDDO-Me inhibited constitutive STAT3 phosphorylation and the degradation of IKBα in ER-negative breast cancer cells, whereas 268 blocked IKBα degradation and the release of interleukin-6 in RAW264.7 macrophage-like cells, inhibited the ability of endothelial cells to organize into networks, and blocked angiogenesis *in vivo*.

**Conclusions:**

CDDO-Me and 268 are useful as individual drugs to prevent ER-negative mammary tumorigenesis and to treat established tumors. They synergize when used in combination for prevention.

In the last several years, the incidence of estrogen receptor (ER)–positive breast cancer has started to decline for women ages >50 years in the United States possibly because of a drop in the use of hormone replacement therapy (1). However, the incidence of ER-negative breast cancer, which frequently occurs in premenopausal women, has not changed significantly in >30 years (1), and >40,000 women still die from breast cancer each year (2). Although chemoprevention will ultimately be needed to meaningfully reduce these mortality rates, new drugs and drug combinations are especially needed for the prevention and treatment of ER-negative breast cancer.

Clinical trials have shown that both selective ER modulators and aromatase inhibitors effectively prevent ER-positive breast cancer in high-risk women (3), but these drugs are ineffective for preventing ER-negative breast cancer. However, the retinoid 9-*cis*-retinoic acid (4), inhibitors of the epidermal growth factor receptor such as gefitinib (5), and rexinoids including bexarotene (6, 7), LG100268 (268; refs. 8, 9), and NRX194204 (4204; ref. 10) inhibit mammary tumorigenesis in preclinical animal models of ER-negative breast cancer. Rexinoids, which bind only to retinoid X receptors and not to the retinoic acid receptors, regulate complex cell survival and differentiation processes by heterodimerizing with other nuclear receptors. The most selective and potent rexinoids, including 268 and 4204, are especially effective in these animal studies and avoid the cutaneous toxicity of retinoids mediated through retinoic acid receptor signaling (3, 11). Bexarotene is Food and Drug Administration approved for the treatment of cutaneous T-cell lymphoma, and 4204 has been approved to begin phase I clinical trials for treatment of various cancers. Because bexarotene retains some binding to retinoic acid receptors and because of the limited availability of 4204 for preclinical studies, we used 268 in our studies.

The synthetic oleanane triterpenoids, including 2-cyano-3,12-dioxooleana-1,9(11)-dien-28-oic acid (CDDO), CDDO-methyl ester (CDDO-Me), and CDDO-imidazolide, are a promising new class of agents for the prevention and treatment of cancer (11). CDDO and CDDO-Me are currently in phase I/II clinical trials for cancer treatment. These three compounds inhibited proliferation and induced apoptosis of breast cancer cells *in vitro* (12–15), slowed the growth of MDA-MB-468 ER-negative cells in nude mice when combined with tumor necrosis factor–related apoptosis-inducing ligand (15), and reduced the tumor burden of xenografted MCF-7 cells stably transfected with HER-2/*neu* (ErbB2; ref. 14). CDDO-imidazolide reduced the volume of preneoplastic lesions by 85% to 99% in the liver of rats exposed to aflatoxin B_1_ (16), and CDDO-Me decreased tumor burden by >90% in the lungs of A/J mice treated with vinyl carbamate, although the triterpenoids were not administered until 2 weeks after tumor initiation with vinyl carbamate (17). Although effective for prevention of liver and lung cancer in these models, the triterpenoids have not been tested for the prevention of experimental breast cancer. The triterpenoids interact with reactive cysteine thiols on target proteins through Michael addition, which helps explain their multiple biological activities (reviewed in ref. 11). Notably, the combination of a triterpenoid and a rexinoid synergizes *in vitro* when used to induce differentiation or apoptosis in leukemia cells (11), and the combination of multiple drugs that work through different mechanisms offers the best strategy for effective chemoprevention of ER-negative breast cancer (3).

In these studies, we tested whether the combination of the triterpenoid CDDO-Me and the rexinoid 268 would synergize in the prevention and treatment of mammary tumorigenesis in the well-characterized MMTV-neu model of ER-negative breast cancer. Because of the striking success of these *in vivo* studies, we then explored the cell types and signaling pathways targeted by these drugs and the antiangiogenic activity of 268.

## Materials and Methods

### Reagents and *in vitro* assays

The E18-14C-27 cells (7) were provided by Powel Brown (Baylor), and the other ER-negative breast cancer cells and the RAW264.7 macrophage-like cells were purchased from the American Type Culture Collection. Cells were grown in either DMEM (E18 and RAW cells) or DMEM/F-12 (MDA cell lines and SK-BR-3 cells) and 10% fetal bovine serum. Stock solutions of CDDO-Me (18) and 268 (19) were made in DMSO, and appropriate DMSO controls (≤0.1%) were included in all *in vitro* experiments. RAW cells were treated with drugs for 24 h, and interleukin-6 (IL-6) and nitric oxide were measured in the medium with a Quantikine ELISA kit (R&D Systems) or the Griess reaction, respectively. Proliferation was measured 72 h after treatment with a [^3^H]thymidine incorporation assay, and apoptosis was detected using a TACS Annexin V-FITC apoptosis detection kit (R&D Systems) and flow cytometry. Antibodies to ErbB2 (RB-103-PO), phosphorylated STAT3 (9138), IKK (sc-7607), and IKBα (sc-371) were obtained from Lab Vision, Cell Signaling, and Santa Cruz Biotechnology. For the immunoprecipitation experiments, E18 cells were treated with 3 μmol/L biotinylated triterpenoid (20) for 1 h and lysed in 100 mmol/L Tris-HCl (pH 7.4), 1% Triton X-100. Total protein (1 mg) was incubated with 50 μL DynaBeads MyOne Streptavidin T1 (Invitrogen) for 1 h, pelleted, and washed four times with Tris-HCl-1% Triton X-100 buffer using the magnets. Samples were resuspended in 40 μL Laemmli loading buffer, boiled for 5 min to remove bound proteins from the beads, and analyzed by Western blotting. Additional details are provided in the figure legends using published protocols (21–23).

### *In vivo* experiments

All animal studies were done in accordance with protocols approved by the Institutional Animal Care and Use Committee of Dartmouth Medical School. For the prevention studies, when female MMTV-neu mice (ref. 24; Jackson Laboratory) were 10 weeks old, they were fed powdered 5002 rodent chow (PMI Feeds) or this powdered diet containing CDDO-Me (60 mg/kg diet), 268 (20 mg/kg diet), or the combination for 45 weeks and were palpated weekly for tumors. For the treatment studies, mice were fed standard chow until tumors at least 32 mm^3^ in volume (*v* = *lwh* / 2) had formed. Mice were then fed 268 (60 mg/kg diet), CDDO-Me (100 mg/kg diet), or the combination in powdered diet. Tumors were measured weekly with calipers and at the end of 4 weeks were classified as regressing (>50% decrease in tumor volume) or actively growing (>50% increase in tumor volume). Evaluation of transgene expression and terminal deoxynucleotidyl transferase – mediated dUTP nick end labeling (TUNEL) staining in tumors has been described (8, 10). To measure proliferation in the tumors, mice were injected i.p. with bromodeoxyuridine (BrdUrd) in PBS (1 mg/mouse). Two hours later, tumors were harvested, and tumor sections (3-5 tumors per group) were analyzed with a BrdUrd *in situ* detection kit (BD PharMingen).

### Angiogenesis assays

To determine morphogenesis *in vitro*, human umbilical endothelial cells (50,000 per well) were suspended in medium with varying concentrations of 268 (0-1 μmol/L) on top of polymerized Matrigel. Images were recorded 6 h later with an inverted microscope (Leitz DM-IRB) equipped with CCD optics and a digital analysis system. For the *in vivo* Matrigel sponge studies, 268 and a cytokine cocktail (100 ng/mL vascular endothelial growth factor-A, 2 ng/mL tumor necrosis factor-α, and heparin) were added to unpolymerized liquid Matrigel. This cold suspension was then injected s.c. into the flanks of C57/BL6 male mice (Charles River), where it rapidly polymerized into a solid gel. Four days later, the gels were recovered and minced to measure hemoglobin content with a Drabkin reagent kit (Sigma; ref. 25).

### Statistical analysis

**Fig. 1:**
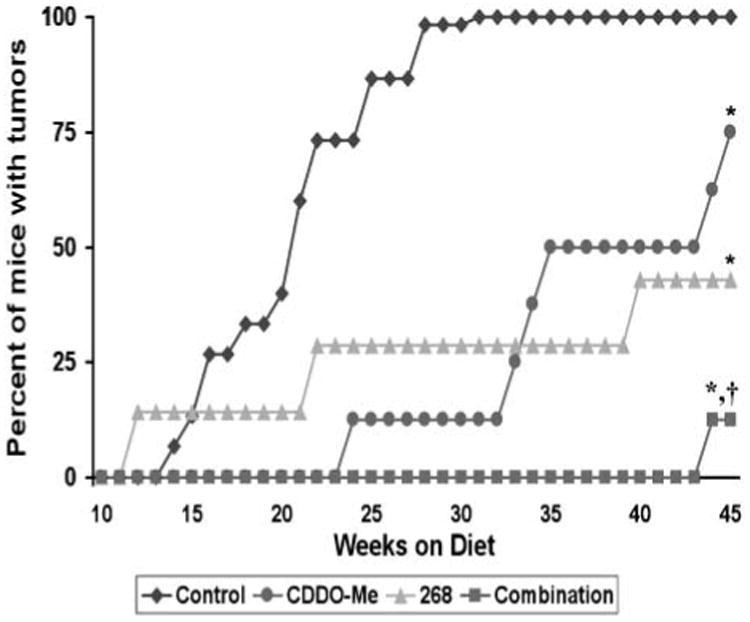
CDDO-Me and 268 delay the development of ER-negative tumors in the mammary gland of MMTV-neu mice. Beginning at age 10 wk, transgenic mice were fed powdered control diet, CDDO-Me (60 mg/kg diet), 268 (20 mg/kg diet), or the combination of CDDO-Me and 268. Mice were palpated weekly, and no tumors were found before the mice were 20 wk old. *n* = 15 mice in the control group and *n* = 8 for all other groups. *, *P* < 0.05 versus control; †, *P* < 0.05 versus CDDO-Me and 268.

Results are mean ± SE and were analyzed either by one-way ANOVA followed by a Tukey test or by one-way ANOVA on ranks (Kruskal-Wallis) and Dunn's method if the data did not pass a normality test (SigmaStat3.5). Percentages were analyzed using a *Z* test and Fig. 1 with a Wilcoxon signed rank test. All *P* values are two-sided.

## Results

### CDDO-Me and 268 synergize for the prevention of ER-negative mammary tumors

**Fig. 2:**
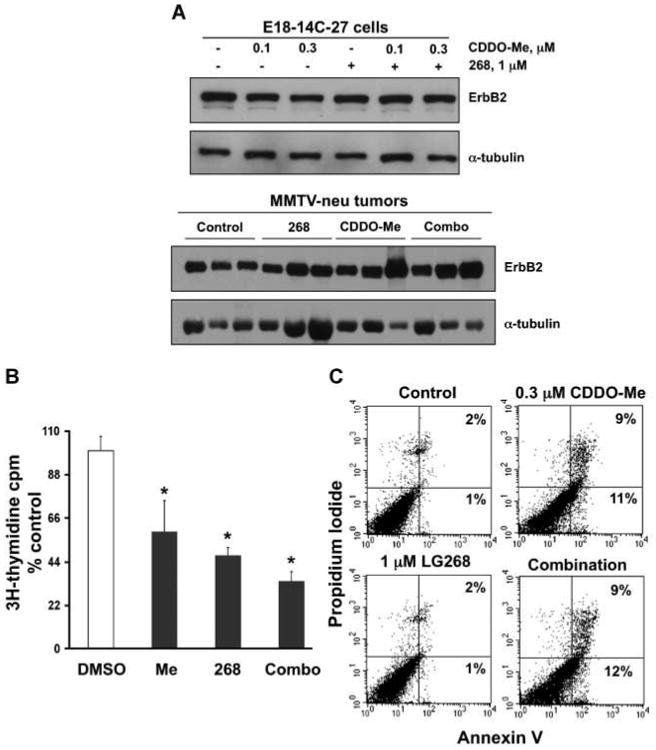
CDDO-Me and 268 do not suppress ErbB2 transgene expression. *A*, E18-14C-27 cells, derived from a MMTV-ErbB2 tumor (7), were treated with compounds for 48 h or MMTV-neu mice with established tumors were fed control diet, CDDO-Me (60 mg/kg diet), 268 (100 mg/kg diet), or the combination for 1 wk, and lysates were analyzed by Western blotting. *B*, E18 cells were incubated with CDDO-Me (0.3 μmol/L), 268 (1 μmol/L), or the combination for 72 h, and cell proliferation was measured with a [^3^H]thymidine incorporation assay. *, *P* < 0.05 versus control. *C*, to examine apoptosis, E18 cells were treated for 48 h, and Annexin V and propidium iodide staining were measured by flow cytometry.

In MMTV-neu transgenic mice, the neu (ErbB2/HER-2) protein, a receptor tyrosine kinase in the epidermal growth factor receptor family frequently overexpressed in human ER-negative breast cancer, is targeted to the mammary gland by the MMTV promoter (24). Beginning at age 10 weeks, these transgenic mice were fed control diet or diet containing the triterpenoid CDDO-Me (60 mg/kg diet or ∼15 mg/kg body weight), the rexinoid 268 (20 mg/kg diet or ∼5 mg/kg body weight), or the combination for 45 weeks. ER-negative mammary tumors were first detected in the control group 10 weeks after starting diet, with 50% tumor incidence by 21 weeks on diet and 100% tumor incidence at 31 weeks on diet (Fig. 1). In contrast, both CDDO-Me and 268 significantly (*P* < 0.05 versus control) delayed the development of these tumors, with 50% tumor incidence occurring at 35 weeks in mice fed CDDO-Me and only a 43% tumor incidence in mice fed 268 for 45 weeks. The combination of CDDO-Me and 268 was significantly (*P* < 0.05 versus CDDO-Me and 268 alone) more effective than either drug alone, as only a single tumor (12.5% incidence) was found in the combination group after 45 weeks on diet. This experiment was repeated with a separate cohort of mice (12 per group) with essentially the same results; each drug alone significantly (*P* < 0.05) delayed tumor development compared with the control group, but the combination of CDDO-Me and 268 was significantly (*P* < 0.05) more potent than each individual treatment (data not shown). These drugs were also well tolerated at these doses, with no evident weight loss or other toxicity. When the mice had been on diet for 24 weeks, the average weight of the mice in the control group was 24.4 ± 3.5 g compared with 24.1 ± 1.4 g in the mice fed CDDO-Me, 28.7 ± 1.7 g in the mice fed 268, and 25.8 ± 0.9 g in the mice fed the combination. These drugs do not inhibit expression of the ErbB2 transgene, as seen in both E18-14C-27 cells, which were derived from a tumor from these mice (7), and mammary tumors treated with CDDO-Me, 268, or the combination (Fig. 2A). Because these compounds also significantly (*P* < 0.05) inhibited the proliferation of E18-14C-27 cells (Fig. 2B) and CDDO-Me induced apoptosis in these cells (Fig. 2C), we tested whether they were effective, either as single agents or in combination, for treatment of established ER-negative tumors in this model.

### Evaluation of proliferation and apoptosis in established ER-negative mammary tumors

**Fig. 3:**
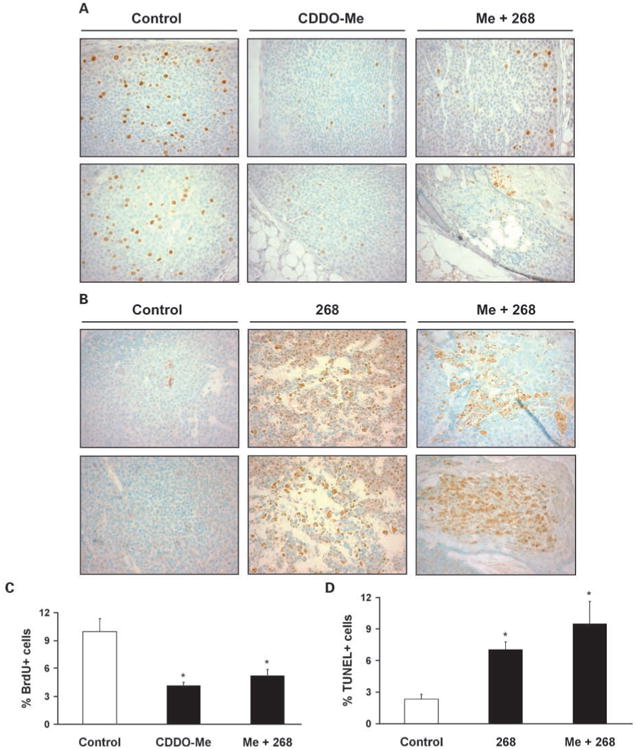
268 increases apoptosis in tumors. Mice were fed powdered diet containing 268 (60 mg/kg diet), CDDO-Me (100 mg/kg diet), or CDDO-Me plus 268 for 1 wk (268) for 2 to 3 wk (CDDO-Me and the combination). Mice were injected with BrdUrd for 2 h, and tumor sections were analyzed by BrdUrd immunohistochemistry (*A*) or TUNEL staining (*B*). Positive cells are stained brown with 3,3-diaminobenzidine; methyl green is the counterstain (magnification, ×400). Mean ± SE of the percentage of BrdUrd-positive (*C*) and TUNEL-positive (*D*) cells from >1,500 cells (4-5 tumors) per treatment group. *, *P* < 0.05 versus control.

In these studies, mice were fed CDDO-Me (100 mg/kg diet or ∼25 mg/kg body weight), 268 (60 mg/kg diet or ∼15 mg/kg body weight), or the combination after they had developed tumors at least 32 mm^3^ in volume. Higher drug doses than those used in our prevention studies were used because it is easier to prevent mammary carcinogenesis than treat advanced breast cancers with their complex and numerous genetic mutations (26). We have reported previously that 268 at 100 mg/kg diet induced regression in established ER-negative tumors in this model and reduced the number of proliferating cell nuclear antigen – positive cells in the tumors (8). When the dose of 268 was lowered to 60 mg/kg diet in these experiments, this dose was still exceptionally potent (Table 1), as it caused a reduction in tumor volume of >50% in 85% of treated tumors (*P* < 0.001 versus control). The growth of the other 15% of the tumors was arrested; none of these tumors continued to grow. CDDO-Me induced regression in 22% of treated tumors and arrested the growth of 64% (*P* < 0.05 versus control) of these tumors. The combination of CDDO-Me and 268 was not better than 268 alone, as 81% of tumors regressed. Although CDDO-Me was not as effective as 268 for inducing tumor regression or apoptosis (data not shown), the tumors did not continue to grow, and BrdUrd immunohistochemistry (Fig. 3A) showed a significant (*P* < 0.01) decrease in the percentage of proliferating cells (BrdUrd-positive cells) from an average of 10.0 ± 1.4 in control tumors to 4.1 ± 0.4 in the CDDO-Me group and 5.2 ± 0.7 in the combination group (Fig. 3C). Treating tumors with 268 or the combination of CDDO-Me and 268 also induced apoptosis, as determined by TUNEL staining (Fig. 3B), and the percentage of TUNEL-positive cells in the tumors significantly (*P* < 0.05) increased 3- to 4-fold from 2.3 ± 0.5 in the controls to 7.0 ± 0.7 for the 268 group and 9.5 ± 2.1 in the combination group (Fig. 3D).

### CDDO-Me and 268 target different proteins and cell types

**Fig. 4:**
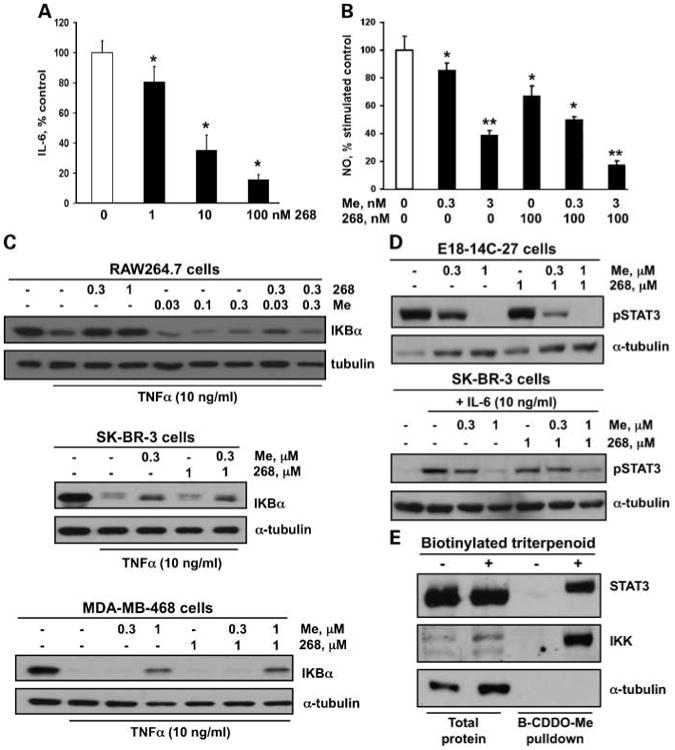
CDDO-Me and 268 affect different cell types and signaling pathways. *A*, RAW mouse macrophage-like cells were treated with 268 and lipopolysaccharide (5 ng/mL) for 24 h, and the amount of IL-6 released into the medium was measured with an ELISA. *B*, RAW cells were treated with CDDO-Me, 268, or the combination and with 10 ng/mL lipopolysaccharide for 24 h, and nitric oxide in the medium was measured by the Griess reaction. *, *P* < 0.05 versus control; **, *P* < 0.05 versus control and individual treatments. *C*, cells were treated with CDDO-Me and 2 68 for 6 h (468 cells) or 24 h (all others), stimulated with tumor necrosis factor-α for 15 min, and lysates were immunoblotted with IKBα antibodies. *D*, ER-negative breast cancer cells were treated with drugs for 24 h, and cell lysates were immunoblotted with antibodies against phosphorylated STAT3 (*pSTAT3*). In E18-14C-27 cells, STAT3 phosphorylation is constitutive; in SK-BR-3 cells, STAT3 phosphorylation was induced by IL-6. *E*, E18-14C-27 cells were treated with a biotinylated triterpenoid (*B-CDDO*-*Me*;3 μmol/L) for 1 h, triterpenoid-protein complexes were precipitated from cell lysates with streptavidin DynaBeads, and IKK and STAT3 were detected by Western blot.

**Fig. 5:**
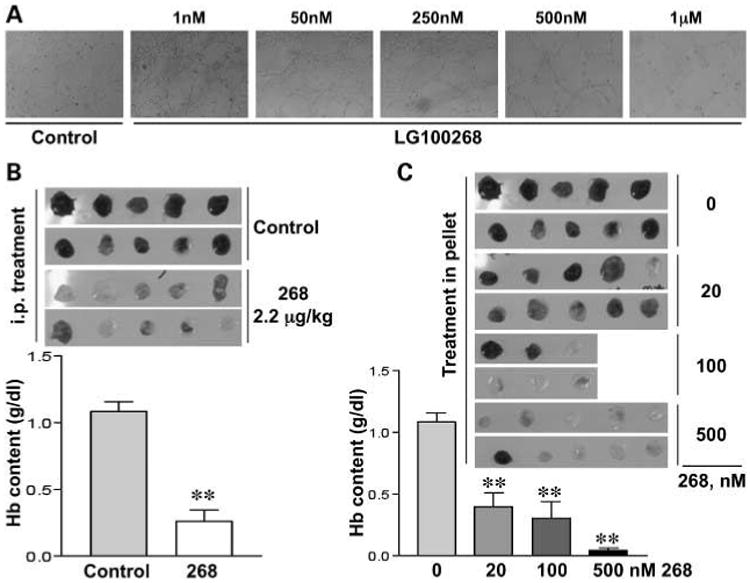
268 inhibits angiogenesis *in vitro* and *in vivo*. Human umbilical vein endothelial cells were plated on Matrigel together with increasing concentrations of 268, and images were captured 6 h later (*A*). Liquid Matrigel was mixed with vascular endothelial growth factor and tumor necrosis factor-α and then injected s.c. into the flanks of mice. 268 was either administered i.p. on day 0 and again on day +2 *B*) or mixed in with the liquid Matrigel (*C*). After 4 days, the solidified Matrigel was removed and the hemoglobin content was measured (g/dL). *n* = 8 mice per group. **, *P* < 0.005 versus control.

Although the rexinoid 268 is exceptionally potent *in vivo* (Fig. 3; Table 1), it does not induce apoptosis *in vitro* (Fig. 2C) of E18-14C-27 epithelial cells, derived from a mammary tumor from these mice, suggesting that 268 may target cells in the tumor microenvironment. Indeed, 268 inhibited the release of IL-6 in a dose-dependent manner in macrophage-like RAW cells stimulated with lipopolysaccharide (Fig. 4A), whereas the triterpenoid CDDO-Me had no inhibitory effect on IL-6 release (data not shown). Both 268 and CDDO-Me inhibited the release of nitric oxide in RAW cells, but the combination of 268 and CDDO-Me was even more potent (Fig. 4B; *P* < 0.05 versus 268 or CDDO-Me alone). Interestingly, pretreatment with 268 prevented the degradation of IKBα induced by tumor necrosis factor-α in RAW cells but not in the SK-BR-3 or MDA-MB-468 ER-negative breast cancer cells (Fig. 4C). In contrast, CDDO-Me prevented IKBα degradation in all ER-negative cell lines, including the E18-14C-27 cells (data not shown), but not in the RAW macrophage-like cells. CDDO-Me also inhibited constitutive phosphorylated STAT3 expression in E18-14C-27 and MDA-MB-468 cells (data not shown) or STAT3 phosphorylation induced by IL-6 in SK-BR-3 cells, but 268 had no effect on phosphorylated STAT3 expression in these ER-negative breast cancer cells (Fig. 4D). The triterpenoids directly interact with IKK and STAT3 in the E18 cells (Fig. 5D) as shown using a biotinylated triterpenoid and streptavidin precipitation with DynaBeads (23). STAT3, the nuclear factor-κB pathway, nitric oxide, and IL-6 are emerging as important targets in breast cancer and the tumor microenvironment (27–35).

### 268 inhibits angiogenesis *in vitro* and *in vivo*

We have reported recently that CDDO-Me blocked angiogenesis and the growth of Kaposi's cells in nude mice (25), and bexarotene reduced angiogenesis in gelfoam sponges and inhibited the migration of human umbilical vein endothelial cells through Matrigel (36). Because of the importance of targeting inflammatory angiogenesis for the prevention and treatment of cancer (37), we examined the effects of the rexinoid 268 on the organization of endothelial cells and their ability to form new blood vessels. Human umbilical vein endothelial cells were cultured on a thick Matrigel layer, which causes the cells to organize into “capillary-like” structures, mimicking the events that occur *in vivo* during angiogenesis. As shown in Fig. 5A, the two highest concentrations of 268, 0.5 and 1 μmol/L, blocked the formation of the “capillary-like” network. 268 is even more potent in the *in vivo* Matrigel sponge model of angiogenesis. In this model, a cocktail of vascular endothelial growth factor and tumor necrosis factor-α induces a potent angiogenic reaction, but 268 either injected into the mouse i.p. (2.2 μg/kg; Fig. 5B) or included in the liquid Matrigel (Fig. 5C) also significantly (*P* < 0.005) inhibited angiogenesis at all concentrations tested.

## Discussion

These studies show that the triterpenoid CDDO-Me and the rexinoid 268 significantly reduced the formation of ER-negative tumors in MMTV-neu mice and that the combination was synergistic for the prevention of mammary tumorigenesis in this model. CDDO-Me targeted IKK and phosphorylated STAT3 and induced apoptosis in ER-negative breast cancer epithelial cells, 268 blocked IKBα degradation and the release of IL-6 in RAW macrophage-like cells but had little effect on breast epithelial cells *in vitro*, and the combination was more effective than the individual drugs for inhibiting the production of nitric oxide in RAW cells. These multifunctional drugs clearly target different signaling pathways in different cell types, an important goal for combination chemoprevention (3, 11).

CDDO-Me reportedly induces apoptosis in a variety of human ER-negative breast cancer cells *in vitro* and in nude mice (11). Although the triterpenoid CDDO-Me, but not the rexinoid 268, induced apoptosis (Fig. 2C) of the E18 breast cancer cell line isolated from a tumor from these mice (7), 268 was significantly more potent for inducing apoptosis and the regression of established ER-negative tumors than CDDO-Me (Fig. 3; Table 1). Several explanations are possible for this seeming discrepancy. First, the dose of CDDO-Me (100 mg/kg diet) used for treating established tumors may not have been high enough to induce apoptosis *in vivo*, because higher concentrations of CDDO-Me are needed to induce apoptosis of cancer cells than to inhibit their proliferation (11). CDDO-Me significantly decreased the number of BrdUrd-positive proliferating cells in tumors (Fig. 3) and either arrested the growth of 64% of tumors in MMTV-neu mice or reduced the volume in 22% of the tumors (Table 1). We did not increase the dose of CDDO-Me used for treatment in the MMTV-neu mice, because higher doses can cause toxicity in mice. This toxicity in rodents at very high doses is not seen in nonhuman primates, as >5 μmol/L drug was detected in several tissues, including the breast, with no observable toxicity or histopathologic findings after the nonhuman primates were given 150 mg drug/kg body weight daily for 14 days by oral gavage (38). In a phase I clinical trial, CDDO-Me was well tolerated in patients at doses up to 900 mg/d for up to 12 months (39), which is much higher than the doses of CDDO-Me used in these studies.

In addition to drug levels, the role of the tumor microenvironment (35) for cancer prevention and treatment needs to be explored. Emerging preclinical and clinical studies suggest that tumor-associated macrophages promote tumor initiation, progression, and metastasis in breast cancer by infiltrating into a tumor and secreting several proangiogenic factors that flip the “angiogenic switch.” The activation of angiogenesis drives the development of a complex vasculature that provides nutrients, oxygen, and growth factors for the growing tumor (40–44). In humans, macrophage “hotspots” in breast cancer samples correlate with high angiogenic activity and poor overall survival (45). In the extremely aggressive polyoma middle T oncoprotein model of ER-negative breast cancer (46), mammary tumors develop rapidly and macrophages infiltrating into the primary tumor activate angiogenesis, drive tumor progression, enhance malignancy, and induce metastases (44, 47). Both CDDO-Me and 268 target macrophages, fibroblasts, and endothelial cells (Figs. 4 and 5; refs. 11, 12, 25, 48, 49), and we are beginning studies to determine whether these drugs or the drug combination can prevent tumor development in the polyoma middle T oncoprotein model by blocking the proinflammatory and proangiogenic activities of macrophages and stromal cells.

Both triterpenoids and rexinoids are multifunctional drugs that target multiple cells and signaling pathways. We have shown that they can synergize for the prevention of experimental ER-negative breast cancer, and with optimization of dose and scheduling, they should synergize for the treatment of breast cancer as well. Although 268 is not being tested clinically, the related and equally potent rexinoid 4204 (10) is entering phase I trials, and the triterpenoid CDDO-Me is already in phase I and II clinical trials for the treatment of cancer. Our data suggest that CDDO-Me and a rexinoid, either 268, 4204, or a new rexinoid, should be tested alone and in combination for the prevention and treatment of breast cancer.

## Figures and Tables

**Table 1:** 268 induces regression of ER-negative mammary gland tumors in MMTV-neu transgenic mice

	Control	268 (60 mg/kg diet)	CDDO-Me (100 mg/kg diet)	268 + CDDO-Me
No. mice in treatment protocol	10	26	11	14
No. tumors in treatment protocol	10	34	14	16
No. tumors with >50% reduction in tumor volume	0	29 (85)*	3 (22)	13 (81)*
No. tumors with arrested growth (%)	0	5 (15)	9 (64)†	1 (6)
No. tumors with active growth (%)	10 (100)	0 (0)*	2 (14)*	2 (13)*
No. tumors not detectable at necropsy, complete regression (%)	0	5 (15)	1 (7)	4 (25)

NOTE: When tumors in female MMTV-neu mice grew to at least 32 mm^3^ in volume, the mice were fed control diet, 268, CDDO-Me, or the combination of 268 and CDDO-Me in diet for up to 4 weeks. Tumors were measured weekly with calipers, and tumor regression was defined as a >50% decrease in tumor volume. An increase in tumor volume of >50% was classified as active tumor growth, whereas growth-arrested tumors did not increase or decrease in size >50% over a 4-week period.

* *P* < 0.001 versus control.

† *P* = 0.006 versus control.
